# The Composition, Antioxidant and Antibacterial Activity of Essential Oils from Five Species of the Magnoliaceae Family

**DOI:** 10.3390/molecules29215182

**Published:** 2024-11-01

**Authors:** Dandan Yang, Daocheng Ma, Ziqi Song, Mei Yang, Yuanyuan Xu

**Affiliations:** Guangxi Colleges and Universities Key Laboratory for Cultivation and Utilization of Subtropical Forest Plantation, Guangxi Key Laboratory of Forest Ecology and Conservation, College of Forestry, Guangxi University, Nanning 530004, China; dd980726@163.com (D.Y.); madaochengmail@163.com (D.M.); 2209302020@st.gx.edu.cn (Z.S.)

**Keywords:** *Micheli*, *Manglietia*, sesquiterpenes, antioxidant activity, antibacterial activity

## Abstract

The leaves of Magnoliaceae family plants contain abundant essential oils (EOs), and these species can be used in many fields due to their high industrial, medicinal, and ornamental values. This study aims to identify the main compounds of the EOs from the leaves of five common Magnoliaceae species (*Michelia maudiae*, *Michelia hedyosperma*, *Michelia macclurei*, *Manglietia lucida*, *Manglietia conifer*) using hydrodistillation, GC–MS analysis, and in vitro tests. Additionally, the antioxidant and antibacterial activities of the EOs were also evaluated. The results show that 151 compounds were identified across five species, with sesquiterpenes being dominant. Some key compounds (such as β-caryophyllene, δ-amorphene, β-guaiene, globulol, and β-acorenol) were common among all the species, highlighting their crucial roles in plant physiology and resilience. Other compounds (like valeranone and nerolidol in *M. maudiae* and β-elemene in *M. macclurei*) were specific, indicating different functions. Among the five species, the essential oil of *M. macclurei* contains β-elemene and nerolidyl acetate, and it has the weakest antioxidant activity (IC50 value: 2918.61~21,341.98 μg/mL) but the strongest antibacterial activity (inhibition zone diameter: 8.55 ± 0.93~22.92 ± 0.46 mm; LC50 value: 0.02~0.78 mg/mL). Meanwhile, the EO of *M. maudiae* demonstrated the best antioxidant activity (the IC50 value was 1283.58~6258.32 μg/mL) and the second-best antibacterial activity (the inhibition zone diameter ranged from 7.61 ± 0.02 to 26.92 ± 0.46 mm, and the LC50 value was 0.03~2.28 mg/mL). Overall, the EO of *M. maudiae* had the best comprehensive performance. Therefore, the EOs of *M. macclurei* and *M. maudiae* showed different performances in biological activity categories, and they could be developed and used in different fields, with the possibility of discovering new applications. This brings inspiration to the potential commercial and industrial uses of sesquiterpenes in Magnoliaceae.

## 1. Introduction

Magnoliaceae is one of the oldest groups of angiosperms, with a fossil record dating back 140 million years to the Cretaceous period [1]. This family is primarily distributed across temperate, sub-tropical, and tropical regions from East to Southeast Asia and encompasses at least 17 genera and 300 species [2]. Its flowers are not only visually striking but also aromatic. The leaves have different shapes and are rich in essential oils (EOs), making them ideal for use as ornamental and therapeutic trees in forest parks and urban environments [3,4]. According to ethnomedicinal practices in China, the U.S., and Mexico, extracts from Magnoliaceae plants contain a large number of sesquiterpenes, which are extremely pharmacologically active and can be used to treat conditions such as rheumatism, anorexia, dysentery, and other diseases [5]. Recent studies have shown that certain compounds in Magnoliaceae EOs have exhibited different pharmacological effects; for example, *Illicium verum* Hook.f. contains trans-anethole, which enhances amylase activity, while anisaldehyde demonstrates antimicrobial activity [6]. The classification of plants within the Magnoliaceae family is diverse and complex. Among its main genera, the EOs of *Michelia* and *Manglietia* are typically rich in sesquiterpenes and oxygenated sesquiterpenes, yet the specific active compounds differ significantly [7,8]. This diverse chemical composition not only underscores the rich pharmacological potential of the Magnoliaceae family but also offers valuable insights for its botanical classification and broader applications.

Antioxidant and antibacterial properties are the main functions of plant EOs, which have showed significant impacts on human health, food safety, and the sustainable development of agriculture [9]. A variety of compounds with antioxidant and antibacterial activities can be extracted from the EOs of Magnoliaceae.

For example, four kinds of terpenoids (linalool, α-terpineol, β-pinene, and geraniol) can be primarily extracted from the EOs of *Michelia alba* DC for use in antioxidant and cardiovascular drugs [10], and lignans are abundant in *Schisandra chinensis* (Turcz.) Baill. EOs have antioxidant effects and can reduce oxidative stress [11], while linalool, enriched in the EOs of *Magnolia sirindhorniae* Noot. and Chalermglin, can inhibit the reproduction of *Staphylococcus aureus* [12]. Additionally, the EOs from the leaves and twigs of *Magnolia hypolampra* (Dandy) Figlar in Vietnam, with α-pinene, β-pinene, and germacrene D as the main constituents, exhibit strong inhibitory effects against *Staphylococcus aureus*, *Escherichia coli*, and *Candida albicans* yeasts [13]. In summary, terpenoids predominate in the EOs of most Magnoliaceae species, providing potent antioxidant and antimicrobial activities.

Analyzing the products of biosynthetic pathways in plant EOs allows for a better understanding of how antioxidant, antibacterial, and other functional compounds are produced. For example, a study found that activating specific signaling molecules can enhance the expression of genes encoding key enzymes in the sesquiterpene biosynthesis pathway, thereby promoting the formation of sesquiterpenes with antioxidant activity in *Atractylodes lancea* [14]. The terpenoids in the EOs of *Michelia* and *Manglietia* exhibit significant biological activities and hold great potential for antioxidant and antibacterial applications. Comparing the composition and activity of these EOs can lead to the identification of tree species more suitable for production practices and lay the foundation for the sustainable use of plant resources. Research has shown that there are not only differences in the content of active ingredients among different medicinal plants [15] but also in the classes of low molecular weight compounds with potential biological activity [16]. Therefore, in addition to terpenoids, comparing potential compounds in *Michelia* and *Manglietia* EOs would also be beneficial for resource development.

In this study, the EOs from the leaves of *Michelia maudiae* Dunn (*M. maudiae*), *Michelia hedyosperma* Y. W. Law (*M. hedyosperma*), *Michelia macclurei* Dandy (*M. macclurei*), *Manglietia lucida* B. L. Chen and S. C. Yang (*M. lucida*), and *Manglietia conifer* var. chingii (Dandy) V. S. Kumar (*M. conifera*) were extracted and analyzed using GC–MS. The best tree species with the highest utilization values were identified based on the antioxidant and antibacterial activities of their EOs. This study addressed the following questions: (1) What are the main characteristics and compounds of the EOs from these five Magnoliaceae species? (2) What are the differences in their antioxidant and antibacterial activities? (3) Which Magnoliaceae species exhibits the highest antioxidant and antibacterial activities? The findings of this study could enhance understanding of the compounds, functions, and applications of Magnoliaceae species EOs and can provide a theoretical basis for their production and broader extension.

## 2. Results

### 2.1. GC–MS Analysis

**Figure 1:**
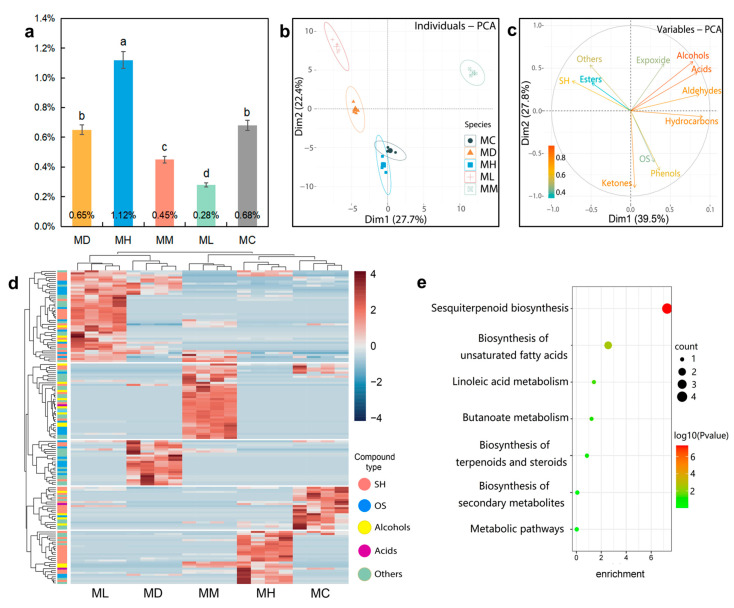
Overview of metabolites the analysis detected in the EOs in five Magnoliaceae plants. (**a**) EO content in leaf extracts of five Magnoliaceae species. Different lowercase letters in the bar charts represent significant differences (*p* < 0.05). (**b**) Principal component analysis of different Magnoliaceae species. (**c**) The contribution of the variables to the principal compounds. The depth of the color of the arrow line represents the degree of contribution. (**d**) Clustering analysis of EO constituents. High and low expression levels of metabolites are shown in red and blue, respectively. (**e**) Bubble chart of 7 signaling pathways in KEGG enrichment analysis. SH: Sesquiterpene hydrocarbons; OS: oxygenated sesquiterpenes. MD: *Michelia maudiae*; MH: *Michelia hedyosperma*; MM: *Michelia macclurei*; ML: *Manglietia lucida*; MC: *Manglietia conifera*.

The EOs’ composition and corresponding contents of the five Magnoliaceae can be seen in Supplementary Table S1. The EO content of the five Magnoliaceae species ranged from 0.28% to 1.12%. (Figure 1a). A total of 151 compounds were identified in the EOs of five Magnolia species and classified into at least 10 groups, including sesquiterpene hydrocarbons, oxygenated sesquiterpenes, epoxide, hydrocarbons, alcohols, phenols, ketones, aldehydes, acids, esters, and others (Supplementary Table S1). Among all the compounds, β-caryophyllene (0.02~31.10%) and δ-amorphene (0.33~39.70%) were dominant. The EOs contained a large number of sesquiterpenes (13.45~86.27%) and oxygenated sesquiterpenes (4.42~74.37%). The main chemical constituents of the EOs varied among the five Magnoliaceae species. For instance, the primary constituent in *M. maudiae* was valeranone (19.39~40.23%), while in *M. hedyosperma*, it was δ-amorphene (17.49~39.70%). In *M. conifera*, β-caryophyllene (10.28~31.99%) was the dominant compound, whereas in *M. macclurei*, it was β-eudesmol (11.30~13.85%). In *M. lucida*, neointermedeol (7.75~10.30%) was identified as the main constituent.

To explain the variance in the compounds of EOs, PCA plots were created based on the content of 151 individual compounds (Figure 1b), and the content of 11 compound classes (Figure 1c) were plotted. In Figure 1b, the first and second principal compounds accounted for 27.7% and 22.4% of the variance, respectively, totaling 50.1%. These results indicate that the differences between the biological replicates of each species were minimal, while the samples of different species were more distinct. Specifically, *M. conifera* and *M. hedyosperma* overlapped, suggesting that their EO compound contents were similar, aligning with the cluster analysis (Figure 1c). In contrast, *M. maudiae*, *M. lucida*, and *M. macclurei* were more distinct from one another, indicating greater differences in their EO compound contents. In Figure 1b, the first and second principal compounds accounted for 39.5% and 27.8% of the variance, respectively, totaling 67.3%. The results reveal that alcohols, acids, and hydrocarbons, located in the first quadrant with the longest line segments, contributed the most to the variance in the first and second principal components. These compounds were key factors in distinguishing the EOs of leaves from different Magnoliaceae species.

To further investigate the metabolic differences of EOs, the relative contents of 151 metabolites were clustered and analyzed (Figure 1d). The EOs of these tree species were categorized into two main clusters on the horizontal axis, while the compounds were grouped into five clusters on the vertical axis. *M. lucida* and *M. maudiae* were classified into cluster I, whereas *M. macclurei*, *M. hedyosperma*, and *M. conifera* fell into cluster II, with *M. hedyosperma* and *M. conifera* being more closely related. In terms of the compound types, *M. lucida* and *M. maudiae* were grouped together, likely due to their higher terpene content and lower overall compound diversity (Supplementary Table S2). On the other hand, *M. hedyosperma* and *M. conifera* were clustered together because they contain not only terpenes but also a higher abundance of acids and other compounds. *M. macclurei* stands out from the other four species, as it is rich in alcohols and acids that are absent in the other species, contributing to its highest compound diversity.

After importing 151 compounds into the pathway database, the resulting enrichment bubble diagram (Figure 1e) revealed five metabolic pathways in the EOs: sesquiterpenoid biosynthesis, biosynthesis of unsaturated fatty acids, linoleic acid metabolism, butanoate metabolism, and biosynthesis of terpenoids and steroids. The differences in the sesquiterpene biosynthesis pathway were the most pronounced, suggesting that sesquiterpene compounds play a crucial role in the cellular metabolism of Magnoliaceae.

### 2.2. Components Identification

**Figure 2:**
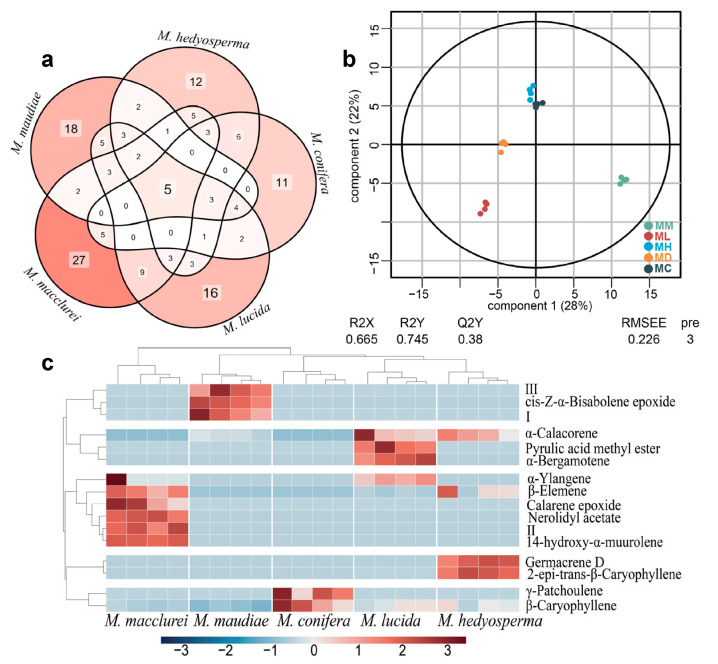
Selection and analysis of DAMs for EOs from five tree species. (**a**) Venn diagram of compounds in different tree species. (**b**) PLS_DA analysis of the EOs composition in five Magnoliaceae species. (**c**) Clustering analysis of the DAMs of EOs in five Magnoliaceae species. High and low expression levels of metabolites are shown in red and blue, respectively. MD: *Michelia maudiae*; MH: *Michelia hedyosperma*; MM: *Michelia macclurei*; ML: *Manglietia lucida*; MC: *Manglietia conifera*. I: 3-buten-2-one, 4-(2,5,5-trimethyl-3,8-dioxatricyclo [5.1.0.0(2,4)]oct-4-yl)-, [1α,2α,4α(E),7α]-; II: 2-[(1S,3Z,7E)-4,8-dimethyl-3,7-cyclodecadien-1-yl]-2-propanol; III: 1,4-methanoazulen-7-ol,decahydro-1,5,5,8a-tetramethyl-, (1S,3aR,4S,7R,8aS)- (8CI,9CI).

Among the five Magnoliaceae tree species, *M. macclurei* had the most specific compounds (totaling 27), followed by *M. maudiae* (totaling 18), and *M. lucida* (totaling 16). The Venn diagram below illustrates the distribution of compounds among these species, with each having its own unique compounds (Figure 2a). Additionally, five compounds are commonly distributed in all five tree species (β-caryophyllene, δ-amorphene, β-guaiene, globulol, and β-acorenol) (Supplementary Table S3).

The analysis of all the compound contents was conducted through a PLS-DA analysis (VIP > 1) to identify 79 metabolites (Figure 2b). Subsequently, parallel sets were taken by two-by-two comparative *t*-test analysis, and 16 DAMs were screened (*p* < 0.05) and then analyzed for cluster analysis (Figure 2c). The PLS-DA model has R2X and R2Y larger than 0.5, but Q2 smaller than 0.05, which suggests that the PLS-DA model performed a reasonably accurate fit. From the clustering results, the DAMs enriched in *M. macclurei* were β-elemene, calarene epoxide, nerolidyl acetate, Ⅱ, and 14-hydroxy-α-muurolene. *M. maudiae* was enriched with Ⅲ, cis-Z-α-bisabolene epoxide, and Ⅰ. *M. conifera* was enriched with γ-patchoulene and β-caryophyllene, which are sesquiterpene hydrocarbons. *M. lucida* was enriched with α-calacorene, pyrulic acid methyl ester, and α-bergamotene. *M. hedyosperma* was enriched with germacrene D and 2-epi-trans-β-caryophyllene, which are sesquiterpene hydrocarbons.

### 2.3. Antioxidant Activities of Different EOs

**Figure 3:**
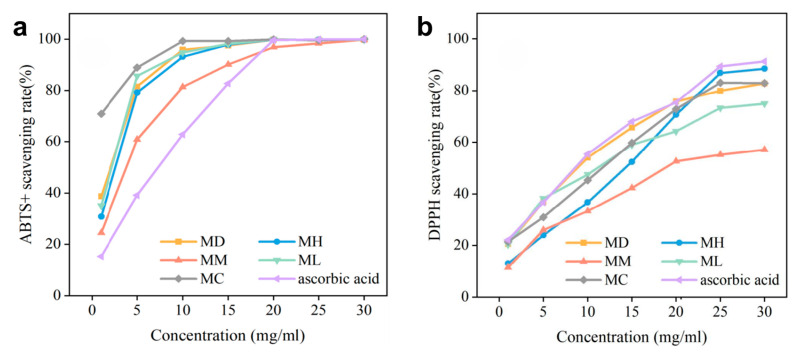
Comparison of antioxidant activities of EOs from five tree species. (**a**) ABTS + scavenging capacity of EOs in five Magnoliaceae plants. (**b**) DPPH radical scavenging capacity of EOs in five Magnoliaceae plants. MD: *Michelia maudiae*; MH: *Michelia hedyosperma*; MM: *Michelia macclurei*; ML: *Manglietia lucida*; MC: *Manglietia conifera*.

Overall, the ascorbic acid and EOs of five tree species were the most effective in clearing ABTS+. The concentration of EOs at 30 mg/mL led to a remarkable scavenging rate of up to 99.98% for ABTS+. In contrast, the scavenging rate for DPPH radicals only reached a maximum of 88.51% (Figure 3). Combining all the results, the antioxidant ability ranked as follows: *M. maudiae > M. conifera* > *M. lucida* > *M. hedyosperma* > *M. macclurei*.

The overall trend of changes in ABTS+ clearance was to increase with an increase in the concentration (Figure 3a, Table 1). The ABTS+ scavenging effects of EOs from all five tree species were better than ascorbic acid. Horizontally, *M. conifera* demonstrates superior ABTS+ scavenging effects compared to the other samples at low concentrations (for example, scavenging rate of 88.93% at 5 mg/mL). *M. maudiae* (81.49% at 5 mg/mL) is in second position, while *M. macclurei* shows the weakest scavenging ability (60.82% at 5 mg/mL). At a high concentration (20 mg/mL), the ATBS+ inhibition was similar in *M. conifera* and *M. maudiae*, both reaching more than 99.8%. In terms of the IC50 values, the ability to scavenge ABTS+ was also best in *M. conifera*, followed by *M. maudiae*. The strongest DPPH scavenger was *M. maudiae*, and the weakest was *M. macclurei*.

The DPPH scavenging rate increases with an increase in the concentration (Figure 3b, Table 1). The ascorbic acid exhibits superior DPPH scavenging at all concentrations compared to the EO. Among the EOs, *M. maudiae* is the best (for example, it has a scavenging rate of 36.86% at 5 mg/mL, and the IC50 value was 6.26 mg/mL), while *M. macclurei* (25.99% at 5 mg/mL, IC50 value was 21.34 mg/mL) shows the weakest performance.

### 2.4. Antibacterial Activities of Different EOs

**Figure 4:**
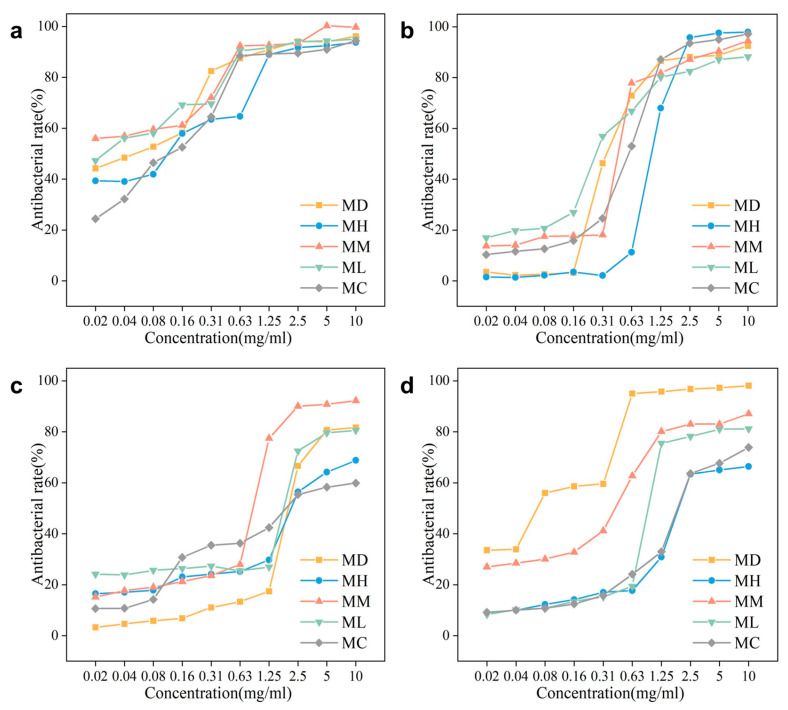
Comparison of antibacterial activities of EOs from five tree species. (**a**) Antibacterial curve of EO against *S. enteritidis*. (**b**) Antibacterial curve of EO against *E. coli*. (**c**) Antibacterial curve of EO against *S. aureus*. (**d**) Antibacterial curve of EO against *B. subtilis*. MD: *Michelia maudiae*; MH: *Michelia hedyosperma*; MM: *Michelia macclurei*; ML: *Manglietia lucida*; MC: *Manglietia conifera*.

Overall (Figure 4, Table 2), *M. macclurei* exhibited the highest diversity of EO compounds (Figure 1d) and demonstrated the most effective inhibitory activity, particularly against *S. enteritidis*, *E. coli*, and *S. aureus*, outperforming the other four species. In contrast, *M. maudiae* showed the strongest inhibition against *B. subtilis* (Figure 4, Table 2).

According to the inhibition of *S. enteritidis* (Figure 4a, Table 2), the highest antimicrobial rate ranged from 93.72% for *M. hedyosperma* to 99.71% for *M. macclurei*. *M. maudiae*, *M. lucida*, and *M. macclurei* exhibited strong inhibition, with inhibition zone diameters ranging from 22.92 ± 0.46 to 26.92 ± 0.46 mm and LC50 values of 0.02~0.03 mg/mL. However, *M. macclurei* was the most effective, achieving an inhibition rate of 55.97% at 0.02 mg/mL, as is evident from the graph.

According to the inhibition of *E. coli* (Figure 4b, Table 2), the highest antimicrobial rate ranged from 88.25% for *M. lucida* to 97.96% for *M. hedyosperma*. *M. macclurei* demonstrated the best inhibition effect, with a relatively high inhibition rate of 94.47% at 10.00 mg/mL, an inhibition zone diameter of 8.55 ± 0.93 mm, and an LC50 value of 0.41 mg/mL. In comparison, the highest inhibition rate of *M. lucida* was 88.25%, and the inhibition zone diameter (6.96 ± 0.40 mm) and LC50 value (1.48 mg/mL) of *M. hedyosperma* were also lower than those of *M. macclurei*.

According to the inhibition of *S. aureus* (Figure 4c, Table 2), the highest antimicrobial rate ranged from 59.92% for *M. conifera* to 92.23% for *M. macclurei*. *M. macclurei* showed the best inhibition. At a concentration of 10.00 mg/mL, and the values of the inhibition zone diameter and LC50 values also performed better than the other four species. Similarly, *M. maudiae* had the optimal performance in all aspects of *B. subtilis* inhibition (Figure 4d, Table 2), with a maximum inhibition of 98.13%, while *M. hedyosperma* was the lowest, with 66.40%.

### 2.5. Correlation Analysis

**Figure 5:**
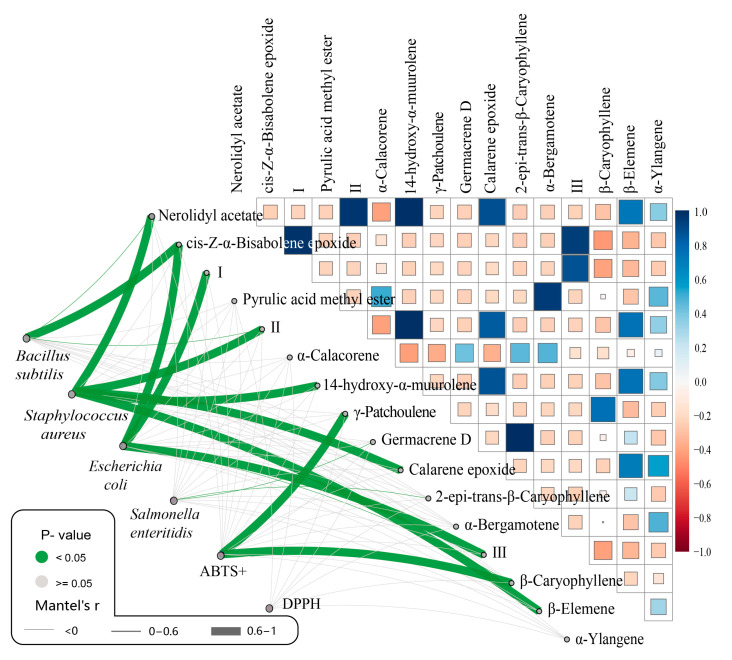
Correlation results of chemical composition and antibacterial and antioxidant activities. Mantel’s r: thicker lines suggest stronger correlations, whereas thinner lines indicate weaker correlations. *p*-value: the color of the lines serves as an indicator of statistical significance. High and low expression levels of metabolites are shown in red and blue, respectively. Mantel’s r > 0.7 indicates a extremely strong correlation. I: 3-Buten-2-one, 4-(2,5,5-trimethyl-3,8-dioxatricyclo [5.1.0.0(2,4)]oct-4-yl)-, [1α,2α,4α(E),7α]-; II: 2-[(1S,3Z,7E)-4,8-dimethyl-3,7-cyclodecadien-1-yl]-2-propanol; III: 1,4-methanoazulen-7-ol,decahydro-1,5,5,8a-tetramethyl-, (1S,3aR,4S,7R,8aS)- (8CI,9CI).

The lines in the left part of the figure below represent the relationship matrix formed by the DAMs and their bioactivities (Figure 5). Combined with Figure 2c, cis-Z-α-bisabolene epoxide was found to be higher in *M. maudiae* and showed significant and positive correlation with the inhibition of *B. subtilis* (*p* < 0.05). Nerolidyl acetate, 2-[(1S,3Z,7E)-4,8-dimethyl-3, 7-cyclodecadien-1-yl]-2-propanol, 14-hydroxy-α-muurolene, calarene epoxide, and β-elemene, five types of compounds highly abundant in the EOs of *M. macclurei*, were strongly correlated with the antagonism against *S. aureus*. Cis-Z-α-bisabolene epoxide, 3-buten-2-one, 4-(2,5,5-trimethyl-3,8-dioxatricyclo [5.1.0.0(2,4)]oct-4-yl)-, [1α,2α,4α(E),7α]-, and 1,4-methanoazulen-7-ol,decahydro-1,5,5,8a-tetramethyl-, (1S,3aR,4S,7R,8aS)- (8CI,9CI), which were highly abundant in the EOs of *M. maudiae*, were strongly associated with the antagonism against *E. coli*. The other two bacteria did not have DAMs with strong correlations. γ-patchoulene and β-caryophyllene, which were abundant in the EOs of *M. conifera*, were strongly correlated with ABTS+ activity, while no DAMs were found to have a strong correlation with DPPH.

## 3. Discussion

### 3.1. Compound Analysis of EOs from Five Magnoliaceae Species

The essential oil extraction times used in this experiment were adjusted based on the results of the pre-experiment. This may lead to different extraction results than those from other studies (e.g., cold pressing or hydrodistillation), especially since some active compounds may be presented in different proportions. For example, the EO compositions of Magnolia maudiae leaves reported from other regions [17] are significantly different from those of the present study, which may be attributed to the effects of abiotic (e.g., experimental methods or environmental changes, such as climate) and biotic factors (e.g., gene expression).

This study shows that sesquiterpenes were dominant in the EOs from the leaves of the five Magnoliaceae species, including β-caryophyllene, δ-amorphene, β-guaiene, globulol, and β-acorenol, which are common among the Magnoliaceae family. These sesquiterpenes are not only present in these species but are also commonly found in other Magnoliaceae plants, such as *Aquilaria crassna* [18], *Piper amalago* [19], and *Iris bulleyana* [20]. The widespread occurrence of these compounds highlights their essential role in regulating seed germination, growth, and pathogen resistance within the Magnoliaceae family [21,22,23]. Among the studied species, *M. macclurei* is particularly interesting due to its significant β-caryophyllene content, which contributes synergistically to the overall efficacy of the plant’s EO [24,25]. Although research on *M. macclurei* is limited, similar studies on other Magnolia species [26] have shown that sesquiterpenes, including caryophyllene and selinene, dominate their EOs. The comprehensive compounds related to the Magnoliaceae and KEGG results (Figure 1e) show that there are commonalities among the five Magnoliaceae and that sesquiterpenes play a key role in the metabolism of Magnoliaceae [27].

Compounds unique to the EOs of each tree species may confer specific ecological or physiological functions, offering unique metabolic pathways or adaptive strategies. For instance, valeranone, an oxygenated sesquiterpene that is abundant in the EOs of *M. maudiae* leaves, aids in environmental adaptation [28]. Additionally, nerolidol, another oxygenated sesquiterpene in *M. maudiae*, serves as a precursor for synthesizing (3E)-4,8-dimethyl-1,3,7-nonatriene, which has an odor that repels animals [29]. Nerolidol is frequently observed in other *M. maudiae* studies [17], indicating it as a chemical characteristic of the species. Similarly, *M. lucida*, a species similar to *M. maudiae* (Figure 1c), contains neointermedeol in its EOs, which exhibits antibacterial and antioxidant activities [30].

Among the identified DAMs, β-elemene in *M. macclurei* stands out due to its notable biological and commercial potential. β-elemene, a key component of traditional Chinese medicine, regulates various molecular targets and shows promising anticancer effects [31]. In *M. macclurei*, β-elemene may accumulate through unique metabolic pathways to become its characteristic metabolite and exert various pharmaceutical benefits. Another important metabolite is cis-Z-α-bisabolene epoxide found in *M. maudiae*. This compound, which may be lost during essential oil extraction, could act as an antioxidant, potentially working synergistically with other compounds [32].

### 3.2. Exploration of the Antioxidant Activity

Antioxidant molecules can inhibit the production of reactive oxygen species by scavenging free radicals generated during cellular metabolism. Therefore, the free radical scavenging rate is often used as one of the criteria for evaluating the antioxidant activity of natural compounds [33]. ABTS+ and DPPH, which are relatively stable organic radicals, are widely used to assess the antioxidant activity of single compounds and different plant extracts [34,35]. Ascorbic acid is commonly used as a standard antioxidant for comparing the antioxidant activity of different samples [36]. In other studies, it has been found that some EOs generally have a lower free radical scavenging capacity than ascorbic acid, a synthetic antioxidant [37,38]. This may be due to the complexity of the composition of EOs, which may contain components that interfere with the antioxidant process, in addition to the antioxidant components. This needs further experimental proof. As shown in Figure 3 and Table 1, the EOs from the five Magnoliaceae exhibited strong scavenging activity against ABTS+ but were weak in scavenging DPPH radicals. This discrepancy might be attributed to the presence of stabilized nitrogen radicals in the DPPH assay, which can lead to an incomplete reaction [39]. Alternatively, the lack of compounds that react with DPPH radicals in the EOs of these Magnoliaceae species [37] could explain the weaker DPPH scavenging ability. Many studies have suggested that phenolic compounds in EOs are essential for antioxidant capacity [34]. However, the EOs of the five Magnoliaceae species had low phenolic content (0.09~0.45%). This phenol, with its two phenolic hydroxyl groups, can trap free radicals and thereby inhibit oxidation reactions.

In this study, β-caryophyllene emerged as both a common compound and a differentially accumulated metabolite (DAM) in the EOs of the five Magnoliaceae species, potentially playing a key role in their antioxidant capacity (Supplementary Table S3, Figure 2). Previous research has shown that β-caryophyllene isolated from the EO of *Aquilaria crassna* possesses antioxidant activity comparable to that of ascorbic acid [18]. In this research, the EOs of *M. conifera* contained the highest amount of β-caryophyllene (10.28~31.99%), which likely contributes to its higher antioxidant activity (IC50 = 69.42~12,279.51 μg/mL). While the EOs of *M. maudiae* contained a smaller amount of β-caryophyllene (0.02~2.80%), they still exhibited antioxidant activity comparable to that of *M. conifera* (IC50 = 1283.58~6258.32 μg/mL). This may be due to the presence of other non-DAMs with strong antioxidant activity properties in *M. maudiae*. Additionally, γ-patchoulene, a sesquiterpenoid commonly found in other plant EOs [40], was present in trace amounts (0.11~0.53%) in the EOs of *M. conifera*. Although γ-patchoulene is rarely mentioned in the context of antioxidant activity, it is necessary to extract it from the EOs of *M. conifera* and test it in vitro to further validate its potential antioxidant effects. Given the results, there is potential for utilizing *M. conifera* and *M. maudiae* as sources of antioxidants or for extracting β-caryophyllene from *M. conifera* to reduce harmful effects, such as those associated with oxidative stress [41]. However, further experiments are required to confirm their practicality as natural medicines.

### 3.3. Exploration of the Antibacterial Activity

Pathogenic microorganisms, such as *Salmonella*, *E. coli*, and *Staphylococcus*, can contaminate food and cause digestive disorders [42]. However, bioactive compounds in EOs can disrupt cell membranes, leading to bacterial death [43]. In Figure 4, the highest inhibition rates of EOs from the five tree species against Gram-positive bacteria (66.4~98.13%) were lower than those against Gram-negative bacteria (88.25~99.71%). This suggests that the EOs of these Magnoliaceae species are more effective at inhibiting Gram-negative bacteria, possibly due to a metabolic system that favors such inhibition. This result is similar to the EOs study of the traditional herb *Cyperus rotundus* L. [44]. Because bacteria such as *S. aureus* are the least sensitive to EOs, they require higher concentrations to be inhibited and killed.

In Figure 5, it can be seen that certain compounds (such as calarene epoxide, β-elemene, nerolidyl acetate, etc.) differed in their effects on Gram-positive and Gram-negative bacteria, which is similar to the results of other studies [45]. However, cis-Z-α-bisabolene epoxide showed significant inhibition of both *B. subtilis* and *E. coli*, indicating it may be a broadly inhibitory compound. The cis-Z-α-bisabolene epoxide may be responsible for the ability of the essential oil of *M. maudiae* to exert better biological activity, though, of course, the effect of other compound interactions cannot be excluded. Similar to this wide range of anti-bacterial effects of essential oil constituents are β-myrcene, borneol, β-farnesene, α-pinene, etc. in *Metasequioa glyptostroboides* Miki ex Hu, which are basically terpenoids [46].

Many of the components of EOs have antioxidant properties, and the synergistic effect of the combination is not the same as that of a single compound. Previous studies using EOs directly for inhibition have found that the inhibitory capacity of EOs is much less than that of artificial agents (Ampicillin, etc.), with minimum inhibitory concentrations hundreds of times greater than those of the agents [13,44,47]. It is only by extracting the active compounds in EOs alone that anti-microbial effects approximating those of artificial agents can be achieved [18]. Therefore, exploring the key active components and exploring the synergistic effects of these substances can help us to better utilize natural EOs. β-elemene, a natural sesquiterpene primarily sourced from *curcuma rhizome*, shows limited antibacterial activity against *S. aureus* (MIC > 1000 μg/mL) [48]. However, this compound was highest in the *M. macclurei* EOs, which had the best *S. aureus* inhibitory ability among the five tree species. (MIC = 780 μg/mL). The correlation analysis in Figure 5 found some antimicrobial correlation for β-elemene but is still limited. It is possible that the synergistic effect of β-elemene and other substances makes *M. macclurei* exert a good inhibitory ability against *S. aureus*, which needs to be proved by further experiments. This indicates that β-elemene may needs to work synergistically with other compounds from *M. macclurei* (like nerolidyl acetate, 2-[(1S, 3Z, 7E)-4, 8-dimethyl-3, 7-cyclodecadien-1-yl]-2-propanol, 14-hydroxy-α-muurolene, and calarene epoxide) to be effective. There are many other minor components of leaf EOs, such as limonene, caryophyllene oxide, and veridiflorol from *Metasequioa glyptostroboides* in one study [46], that may have some antimicrobial synergy with the other active ingredients.

The antibacterial activity of Magnoliaceae EOs is influenced by the types, structures, and functional groups of their compounds. While individual EOs like those from *M. macclurei* or *M. maudiae* may not be sufficient to inhibit multiple bacteria, understanding the interactions between different compounds and exploring their synergistic effects is crucial. Future studies should focus on these interactions and develop efficient extraction processes for antibacterial substances.

## 4. Materials and Methods

### 4.1. Test Site and Materials

(1) Test site: Leaf samples were collected in the suburbs (22°28′ N, 108°18′ E) of Nanning, Guangxi, China. The region experiences a typical subtropical monsoon climate, with an average annual temperature of approximately 21.6 °C, average annual precipitation of 1304.2 mm, and an average relative humidity of 79%. The sampling site is primarily hilly, with altitudes ranging from 150 to 400 m and slopes of 25 to 30°, including semi-shaded, semi-exposed, and sunny slopes. The soil is a typical lateritic red soil, characterized by a thickness of 70 to 120 cm, and acidic topsoil.

(2) Plant materials: The plant species were identified by Ms. Deng Rongyan (teacher of plant taxonomy) from the School of Forestry, Guangxi University. The trees of five Magnoliaceae species (*M. maudiae*, *M. hedyosperma*, *M. macclurei*, *M. lucida*, and *M. conifera*) were planted in 2016 with a spacing of 2 m × 3 m. After transplanting, grass was cut three times per year, and fertilizer was applied once annually from 2016 to 2019. In September 2020, three 400 m^2^ sample plots (20 m × 20 m) were established in the forests for each Magnoliaceae species. In each plot, 3~4 healthy, medium-sized trees were randomly selected. Mature, healthy leaves were collected from the lower and middle part of the canopy and pooled together by species. The plant name has been verified with World Flora Online (www.worldfloraonline.org, accessed on 20 September 2024).

### 4.2. EO Extraction and Compound Identification

(1) EO extractions: The experimental steps were based on the method outlined by Refs. [49,50], with some modifications. We used steam distillation for the extraction of EOs and adjusted the extraction duration according to the pre-experimental results. The leaves from the five species were first washed with clean and deionized water and then dried in an oven. After sterilizing at 110 °C for 30 min, the leaves were further dried at 80 °C until they reached a constant weight. The dried leaves were then crushed and sieved through an 18-mesh screen. Approximately 100 g of the dried leaves were placed in a 1 L round bottom flask with a leaf-to-water ratio of 1:5 and soaked for 12 h. The mixture was hydrodistilled for 10–12 h. After separating the water and oil layers in the reflux tube, the volume of EO was measured. The oil was dried over anhydrous sodium sulfate, and 2 μL was dissolved in n-hexane for GC–MS analysis. The remaining oil was stored at 4 °C. The extraction rates of EOs from Magnoliaceae leaves were calculated by the following formula:

Percentage Yield (%) = (W_E_ ÷ W_S_) × 100

where W_E_ = the weight of the plant extract and W_S_ = the weight of the initial sample.

(2) Composition determination: Four portions of EO from each tree species were subjected to GC–MS analysis. The analysis was performed using a QP5050A gas chromatograph coupled with mass spectrometry (GC–MS) (Shimadzu Corporation, Kyoto, Japan), equipped with a TG-5SIMS capillary column (30 m × 0.25 mm, with a film thickness of 0.25 mm). The GC oven temperature was programmed as follows: initially set at 100 °C for 2 min, then increased to 220 °C at a rate of 4 °C/min, and finally ramped up to 270 °C at a rate of 8 °C/min, where it was held for 10 min. Helium was used as a carrier gas, and high-purity argon was used as a collision gas. The purge flow rate was 5 mL/min; the column flow rate was at a constant flow of 1.0 mL/min; the split ratio was 1:50; and the injection volume was 1 μL.

For mass spectrometry, an electron ionization (EI) ion source was used, scanning a mass range of 33–800 *m*/*z* in full-scan mode with a solvent delay of 3 min. The inlet temperature was set at 270 °C, the transfer line temperature at 280 °C, and the ion source temperature at 300 °C. The constituents of the essential oils were identified by comparing their retention times with those of reference standards, their retention indices relative to the series of n-Hexane, and by comparison of their mass spectra with published spectra (NIST2002). The relative concentration of each compound was calculated by the integration of gas chromatography peak areas.

### 4.3. Antioxidant Assay

(1) ABTS+: The ABTS+ and DPPH scavenging assays were performed according to previously reported protocols, with minor modifications [51]. A 7 mmol/L 2,2′-azinobis-(3-ethylbenzothiazoline-6-sulfonic acid) cation radical solution was mixed with a 2.45 mmol/L potassium persulfate solution at a volume ratio of 1:1. This mixture was allowed to stand for 12~16 h at room temperature in the dark to form the ABTS+ solution. The solution was then mixed with ethanol at a volume ratio of 1:80. The absorbance was measured at 734 nm to achieve a value 0.7 ± 0.02 [52]. The solution was prepared and used immediately. For the assay, 2.5 g of essential oils (EOs) were dissolved in 25 mL of anhydrous ethanol at different concentrations (1, 5, 10, 15, 20, 25, 30 mg/mL) to prepare the samples. Ascorbic acid solutions of varying concentrations (1, 10, 20, 40, 60, 80, 100 mg/mL) were prepared as positive controls. In each test tube, 0.2 mL of each sample (1~30 mg/mL) and positive control (1~100 mg/mL) were added, followed by the addition of 4 mL of ABTS+ solution. The mixtures were thoroughly mixed and kept at room temperature in the dark for 30 min. For the blank control (A0), 0.2 mL of distilled water was used in place of the sample, and for the reference control (A2), 4 mL of distilled water was used in place of the ABTS+ solution. Each treatment was repeated three times, and the scavenging rate was calculated and averaged. The absorbance of A0, A1, and A2 was measured at 734 nm (OD). The scavenging activity (%) was calculated using the following formula:

Scavenging activity (%) = (OD_A0_ − OD_A1_ + OD_A2_/OD_A0_) × 100%

(2) DPPH: An aliquot of 2 mL of 1,1-diphenyl-2-picrylhydrazyl (DPPH) solution (0.1 mM) was mixed with 2 mL of different concentrations of EO (1~30 mg/mL). After being kept in the darkness at room temperature for 30 min, the absorbance was measured at 516 nm [52]. Absolute ethanol and distilled water were used as controls and labeled A0 and A2, respectively. Ascorbic acid was used as a positive control, with concentrations of 1, 10, 20, 40, 60, 80, 100 mg/mL. The calculation formula is the same as the ABTS+ assay. The effect of EO on scavenging ABTS+/DPPH was statistically analyzed using SPSS 27 software. The regression equation was obtained by fitting the bacteriostatic curve, and the half-maximal inhibitory concentration (IC50) was calculated.

### 4.4. Antibacterial Assay

Four common experimental bacterial strains were selected for this study. The Gram-positive bacteria were *Bacillus subtilis* (*B. subtilis*) and *Staphylococcus aureus* (*S. aureus*), while the Gram-negative bacteria were *Escherichia coli* (*E. coli*) and *Salmonella enteritidis* (*S. enteritidis*). All the strains were obtained from the corporation of BeNa Culture Collection (Beijing, China). The EO compounds from the five Magnoliaceae plants were diluted in anhydrous methanol to different concentrations (0.02, 0.04, 0.08, 0.16, 0.31, 0.63, 1.25, 2.50, 5.00, 10.00 mg/mL). The bacteria were subcultured once using the nutrient agar medium (beef extract 3.0 g, peptone 10.0 g, NaCl 5.0 g, agar 20.0 g, distilled water 1.0 L, pH 7.0). The OD600 of the bacterial solution was measured (when *E. coli* OD600 = 0.1, the bacterial concentration was approximately 108 CFU/mL, and other bacteria were referenced to this value). The bacterial solution was diluted to 1 × 105 CFU/mL using a 10-fold dilution with liquid medium (beef extract 3.0 g, peptone 10.0 g, NaCl 5.0 g, distilled water 1.0 L, pH 7.0).

The filter sheet-agar diffusion method was used in this study. After high-temperature sterilization, 15 mL of solid medium was poured into each dish and incubated at a constant temperature for 24 h to ensure no colony growth. The plates were then placed on a super clean bench. A pipette was used to draw 100 μL of the bacterial suspension, which was then injected onto the plates. The suspension was evenly spread using a triangular spreader to create a bacteria-containing plate for later use.

Circular qualitative filter papers, 6 mm in diameter, were sterilized at a high temperature and immersed in the EOs of the five tree species at a concentration of 10 mg/mL for 30 min. The filter paper discs, impregnated with the sample solution, were then attached to the center of the bacteria-containing plates. The plates were placed horizontally for 2 h, followed by incubation in an inverted position in a cell culture incubator for constant-temperature incubation. After 24 h, the diameter of the inhibition zones around the filter papers was measured. Filter papers impregnated with anhydrous methanol were used as the control. Each bacterial strain was tested three times, and the average inhibition zone diameter was recorded.

The filter paper method visualizes the inhibitory effect of EOs on bacterial growth, whereas the resazurin indicator microdilution method reflects the antimicrobial effect of different EO concentrations through a gradient. The resazurin indicator-microdilution method [53] was employed in this study. The resazurin indicator-microdilution method is a microbiological assay. It uses a change in the color of the resazurin indicator to reflect the metabolic activity of microorganisms, thus determining the status of microorganisms in the sample. A sample solution with a concentration gradient of 0.02~10.00 mg/mL was prepared using a liquid medium at a ratio of 1:4 and labeled as A0. Anhydrous methanol was mixed with liquid medium (1:4) to create the methanol–liquid medium. The resazurin indicator was prepared at a concentration of 300 μg/mL. To begin the assay, 50 μL of a 1 × 10^5^ CFU/mL bacterial solution was added to columns 1 through 12 of a 96-well plate. Next, 50 μL of the sample–liquid medium, with 10 concentration gradients, was sequentially added to columns 1 through 10. In column 11, 50 μL of the methanol–liquid medium was added as the sterile control (A1), and in column 12, 50 μL of the liquid medium was added as the drug-free control (A2). The 96-well plate was incubated in a 37 °C constant-temperature shaking incubator for 20 h. After this period, 5 μL of the resazurin indicator was added, and the plate was shaken for an additional 4 h. The OD value was measured at 570 nm to determine the bacteriostatic rate, which was then calculated and averaged.

Bacteriostatic rate = (ODA2 − ODA0)/(ODA2 − ODA1) × 100%

### 4.5. Statistical Analysis

The relative content of each compound was calculated using peak area normalization based on the GC–MS results. Data collection and comparison of EO compounds in the five Magnoliaceae species were conducted using Microsoft Excel 2016 and IBM SPSS Statistics 27. One-way ANOVA and Duncan’s test were used to assess the significant differences (*p* = 0.05). Differentially accumulated metabolites (DAMs) were identified using *t*-test and PLS-DA analysis (VIP > 1, *p* < 0.05) by R4.4.1 [54]. PCA plots, clustering heat maps, a Venn diagram, and correlation heat maps were drawn using R4.4.1. Metabolite functions were annotated based on the KEGG database (www.metaboanalyst.ca, accessed on 20 September 2024).

## 5. Conclusions

The essential oils from the five Magnoliaceae species exhibited variations in compound type and quantity, with terpenoids emerging as the predominant constituents, especially in the metabolic pathway of sesquiterpene biosynthesis. *M. macclurei* was notably rich in β-elemene, nerolidyl acetate, and other compounds, showcasing the best antibacterial capacity. However, when considering both antioxidant and antibacterial strength, the essential oil of *M. maudiae* proved to be more valuable for potential utilization. While β-caryophyllene and δ-amorphene are common in the essential oils of all five species, compounds like cis-Z-α-bisabolene epoxide and β-elemene are unique to certain species. The biological activity of these compounds warrants further investigation. By analyzing these essential oil compounds, this research contributes to the ecological classification of Magnoliaceae species and supports the search for more efficient natural active agents, promoting the rational utilization of forest natural resources.

## Figures and Tables

**Table 1:** IC50 (μg/mL) values of the EOs in five Magnoliaceae plants obtained through different antioxidant mechanisms.

Free Radical	Sample					
	*M-maudiae*	*M-hedyosperma*	*M-macclurei*	*M-lucida*	*M-conifera*	Ascorbic Acid
ABTS+	1283.58	1694.57	2918.61	1387.45	69.42	4761.25
DPPH	6258.32	14,028.60	21,341.98	7988.74	12,279.51	19,486.27

**Table 2:** Comparison of bacteriostatic circle diameter (mm) and LC50 (mg/mL) values of the EOs in five Magnoliaceae plants.

Microbial Strain	Inhibition Zone Diameter (mm)				
	*M. maudiae*	*M. hedyosperma*	*M. macclurei*	*M. lucida*	*M. conifera*
*Salmonella enteritidis*	26.92 ± 0.46 Aa	16.71 ± 0.43 Ad	22.92 ± 0.46 Ab	23.63 ± 0.55 Ab	18.04 ± 0.41 Ac
*Escherichia coli*	7.61 ± 0.02 Cc	6.96 ± 0.40 Bc	8.55 ± 0.93 Db	10.29 ± 0.28 Ba	7.33 ± 0.30 Cc
*Staphylococcus aureus*	7.87 ± 0.27 Cc	6.57 ± 0.37 Bd	15.78 ± 0.18 Ba	6.57 ± 0.37 Dd	8.61 ± 0.12 Bb
*Bacillus subtilis*	15.03 ± 0.74 Ba	6.41 ± 0.46 Be	12.91 ± 0.26 Cb	9.52 ± 0.22 Cc	8.38 ± 0.73 Bd
**Microbial strain**	**LC50(mg/mL)**				
	***M. maudiae***	***M. hedyosperma***	***M. macclurei***	***M. lucida***	***M. conifera***
*Salmonella enteritidis*	0.03	0.09	0.02	0.02	0.11
*Escherichia coli*	0.64	1.48	0.41	0.32	0.67
*Staphylococcus aureus*	2.28	2.62	0.78	1.79	2.28
*Bacillus subtilis*	0.07	2.46	0.28	1.00	2.34

Note: Data represent means ± standard deviations; different lowercase letters in the columns represent significant differences (*p* < 0.05). Different capital letters in the same column indicate significant differences among different bacterial strains (*p* < 0.05), and different lowercase letters in the same row indicate significant differences among different tree species (*p* < 0.05).

## Data Availability

The raw data supporting the conclusions of this article will be made available by the authors on request.

## Supplementary Materials

The following supporting information can be downloaded at: https://www.mdpi.com/article/10.3390/molecules29215182/s1, Table S1: Summary of all compounds; Table S2: Compound Classification; Table S3: Shared and unique compounds; Table S4. PLS_DA; Table S5. *T*-test; Table S6. Antioxidant Trends; Table S7. Antibacterial trends.
